# A comparison of adult-child and spousal cancer caregivers’ participation in medical decisions

**DOI:** 10.1371/journal.pone.0300450

**Published:** 2024-06-13

**Authors:** Anny T. H. R. Fenton, Katherine A. Ornstein, Erin E. Kent, Ellen Miller-Sonet, Alexi A. Wright, J. Nicholas Dionne-Odom

**Affiliations:** 1 Division of Population Sciences, Dana Farber Cancer Institute, Boston, MA, United States of America; 2 Center for Equity in Aging, Johns Hopkins University School of Nursing, Baltimore, MD, United States of America; 3 Gillings School of Global Public Health, University of North Carolina, Chapel Hill, Chapel Hill, NC, United States of America; 4 Lineberger Comprehensive Cancer Center, University of North Carolina, Chapel Hill, Chapel Hill, NC, United States of America; 5 Cancer*Care*, New York, NY, United States of America; 6 Harvard Medical School, Boston, MA, United States of America; 7 School of Nursing, University of Alabama at Birmingham, Birmingham, AL, United States of America; 8 Division of Gerontology, Geriatrics and Palliative Care, School of Medicine, University of Alabama at Birmingham, Birmingham, AL, United States of America; Swiss Paraplegic Research, SWITZERLAND

## Abstract

**Background:**

Family caregivers often play a key role in medical decision-making for patients with cancer. Adult-children account for nearly half of caregivers, but often have less experience with serious illness care and decision-making and face unique relational challenges as the patient’s child. Yet little research explores the potentially distinctive decision-making, involvement in decisions, and support needs of adult-child caregivers.

**Methods:**

Analysis of survey data of U.S. cancer caregivers conducted by Cancer*Care*® in 2021. Chi-square tests and multivariable regression models assessed whether adult-child and spousal caregivers differed on the type of medical decisions they participated in (e.g., treatment planning, medication management), who made the decision (e.g., caregiver or joint decision), and the resources that informed decisions (e.g., friends and family, education materials).

**Results:**

Adult-children (N = 892) were less likely than spouses (N = 314) to participate in treatment planning (beta = -0.41; 95%CI = -0.81,-0.01), but more likely to be involved in decisions about whether to challenge medical authority (e.g., seeking alternative treatment, second opinion) (beta = 0.50; 95%CI = 0.22,0.78). Compared to spouses, adult-children made joint decisions with patients less often (-13.2-percentage points; 95%CI = -19.64,-6.67) and acted as primary decision-maker more frequently (5.60-percentage points; 95%CI = 0.01,10.43). More adult-children than spouses sought help and information regarding decisions from the oncology team (8.42-percentage points; 95%CI = 1.98,14.87) and friends and family (7.91-percentage points; 95%CI = 1.34,14.48).

**Conclusions:**

How cancer caregivers and patients are related to each other shapes caregivers’ medical decision-making. Adult-children’s and spouses’ probabilities of participating in and influencing decisions differed for certain types of decisions while adult-children were more likely to seek information and social support regarding decisions. These findings highlight the importance of the patient’s and caregiver’s relationship type in medical decision-making, suggesting that decision support programs may be more effective if they tailor programs by relationship type.

## Introduction

From the initial diagnosis of cancer, patients face numerous consequential and complex medical decisions, from where to receive treatment, to symptom management, to financial decisions. These medical decisions can be cognitively and emotionally taxing for patients. Unsurprisingly, patients often collaborate with a family caregiver (e.g., family member, friend) to help make these decisions [1]. In one study, over 70% of cancer patients reported that a family member meaningfully participated in their medical decision-making [2]. Including caregivers in decision-making can also be beneficial for patient outcomes; family involvement in decision-making is linked to patients’ satisfaction with care, their increased decision involvement, treatment adherence, and decreased stress along with more goal concordant care [3–6]. Hence, identifying what factors promote caregivers’ involvement is critical to optimizing care.

Despite caregivers’ importance to medical decision-making, little is known about how different social relations (e.g., spouses, siblings) impact medical decision-making. This knowledge gap is potentially problematic since nearly half of cancer caregivers are adult-children caring for their parent, [7] but studies have disproportionately focused on spousal caregivers’ decision-making experiences [8, 9]. Adult-children may face unique challenges compared with spousal caregivers; prior studies show that adult-children have less experience making medical decisions [10] and are less likely to agree with the decisions made [11, 12]. Adult-children may also experience more social, emotional, and financial caregiving burden than spouses, [13] in part because they are also more likely to juggle employment and caregiving than spouses [14]. The caregiver’s social position as the patient’s child may also limit their involvement: many adult-children report struggling with the cultural authority to advise their parents’ choices [15]. Ultimately, these differences may impact the ways that adult-children partner with patients to make medical decisions.

Given gaps in the literature describing how adult children are involved in medical decisions, what resources they rely on for decision-making, and their decision-making experiences, we pursued the following questions:

1. Are adult-children involved more frequently in certain types of medical decisions than spousal caregivers?
2. Does the primary decision-maker(s) differ when an adult-child is the caregiver versus a spouse?
3. Are adult-children and spousal caregivers’ decisions influenced/helped by different individuals and information sources?

We hypothesized that adult-children would be more likely to question medical authority (e.g., seek a second opinion), due to modern shifts towards more patient-centered care [16]. We also expected adult children to be less involved in medical decision-making compared to spouses, and more likely to indicate that the patient or oncology team was the primary decision-maker, due to adult-children’s social position as the patient’s child and (likely) relative inexperience with serious medical care. We further hypothesized that adult children would be more likely to seek out information from friends, family members, and the medical team, compared with spouses, and would feel more positively about the medical decisions made.

## Data & methods

Adult cancer caregivers (N = 2703) across the U.S. completed an online survey in 2021 developed by Cancer*Care*®, a national non-profit that provides free services and resources to patients with cancer and their families. See Cancer*Care* (2022) and Dionne-Odom et al. (2022) for full survey development and item details [17, 18]. Respondents were surveyed about their medical decision-making experiences, details about their caregiving responsibilities, and their demographic characteristics. Caregivers were recruited from authenticated consumer panels of PureSpectrum, Inc., a market research platform. Survey eligibility required that participants currently provide unpaid support to a friend or family member with cancer for at least six months and that they report involvement in at least one health-related decision for the patient. Caregivers from racial and ethnic minority groups were oversampled to ensure a diverse sample [17]. We excluded caregivers who did not identify the patient as their spouse/partner or parent (N = 1497). We also excluded those who used the survey’s free-form entry to identify the patient as a stepparent (N = 1) or parent-in-law (N = 13). We hypothesized that, since these adult-child caregivers specified their relationship with the patient as non-parental, their pattern of involvement may differ from those who identified as caring for a parent. The final cohort included 1,206 caregivers, of whom 74% identified as adult-child caregivers. Cancer*Care*® shared de-identified data with the study team. The study was deemed exempt by the University of Alabama at Birmingham Instutional Review Board.

### Variables

#### Dependent variables

Decisions (Research question 1): Survey respondents were asked about their involvement (yes/no) in fourteen decisions about treatment, medical management, supportive care, and end-of-life decisions.

Decision types (Research question 1): To estimate whether adult-child and spousal caregivers’ involvement differed based on different types of decisions, we categorized the eight most frequently remembered decisions into three broad types (planning treatment, challenging medical authority, assessing time-sensitive medical situation), grouping the remaining six in a catch-all other category. Decisions were categorized based on shared qualities for the types of skills, knowledge, and assessments that we theorized that caregivers would draw on when making these decisions. Dummy variables for each type indicated whether a caregiver participated in any of the associated decisions.

- Planning treatment: ‘Whether to begin treatment,’ ‘The treatment plan (e.g., surgery, radiation, chemo, immunotherapy, targeted therapy),’ ‘Where to get treatment’
- Challenging medical authority: ‘Whether to get a second opinion on the treatment plan,’ ‘Whether to get alternative, non-traditional therapy (such as high dose vitamins and supplements, homeopathy, chelation),’ ‘Whether to switch to another doctor or cancer center’
- Assessing time sensitive medical situation: ‘Determining what medications to take to manage symptoms,’ ‘Deciding if the patient should go to the ED.’
- Non-classified: ‘Whether to be in a clinical trial,’ ‘Whether to get a biomarker test,’ Whether to get palliative care,’ ‘Whether to get rehabilitative services,’ ‘Whether to have hospice.’

The survey further asked respondents to report, of the decisions they participated in, which they ‘remembered the most clearly.’ Respondents were then asked to answer subsequent questions with this ‘remembered’ decision in mind, including the following dependent variables.

Decision-maker (Research question 2): Respondents indicated who was the primary decision-maker (i.e., the caregiver, the patient, the caregiver and patient together, or the clinical team) and could select multiple responses. We created a categorical variable denoting which individual or group was the primary decision-maker. If a respondent marked multiple responses, we coded the primary-decision maker variable as multiple persons. We excluded respondents who selected ‘other’ and were able to enter further explanatory text (n = 21).

Others’ involvement (Research question 3): Respondents could indicate whether the patient, family/friends, a spiritual or religious counselor, the oncology team, or a non-oncology provider were also involved with the most clearly remembered decision. We created a dummy variable for each individual’s or group’s involvement (yes/no).

Sources of information and help (Research question 3): We measured what types of information and help caregivers sought for the remembered decision based on their responses to whether they used the following resources: oncology team, friends/family, non-oncology team medical professionals, internet, social media, patient education from oncology team, government agencies, and caregiving and cancer non-profits, or that they never sought any resources. We classified each potential response as yes/no.

#### Independent variable

Caregiver-patient relationship: We measured this relationship with a dummy variable with spousal caregiver as the reference category.

#### Covariates

We measured several demographic variables including caregiver and patient gender (female, male), age, race (Asian, Black, White, other racial groups), and Hispanic ethnicity (yes, no). We also measured caregiver’s educational attainment (high school/GED or less, some college/Associate degree, 4-year college degree, post-graduate degree), employment (retired/unemployed and not looking for work, part-time employment/student/unemployed and looking for work, full-time employment), and rural residence (based on RUCA zip code collapsed into rural vs. urban) [19]. We also report caregiving factors including patient’s cancer stage and type, the number of hours of care the caregiver provides daily, whether the patient and caregiver live together, and the caregiver’s rating of how effectively the patient could communicate with their oncology doctor (never/not usually/sometimes, most of the time/all of the time). When decision type is used as a covariate (research questions two and three), it is a four-level categorical variable (planning, medical challenge, assessing time-sensitive situational, non-classified).

### Analyses

Chi-square were used to estimate overall differences between adult-child and spousal caregivers in means or proportions for their respective measures across samples. For research questions one and three, we used binary logistic regressions to estimate the probability of each outcome and multinomial logistic regressions for research question two. Multinomial logistic regression coefficients convey the probability of one outcome relative to a reference outcome. However, we were interested in whether an adult-child’s probability of an outcome is different than the spouse’s probability of the same outcome. Thus, to aid interpretation in research question three, we use STATA’s margins command to estimate adult-children’s versus spouses’ probability of the outcome (see Table 1 for details) [20].

**Table 1:** Analyses details by research question.

Research question	Regression model	Survey item	Difference by decision type analysis	Results
1: Are adult-children involved more frequently in certain medical decisions than spousal caregivers?	Binary logistic	Response for each of the 14 decisions	Estimate if Y is decision type or not	Log-odds
2: Does the primary decision-maker(s) differ when an adult-child is the caregiver versus a spouse?	Multinomial logistic	Select 1 of 14 decisions to base response on	Interaction term: caregiver relationship x decision type	Difference in predicted probability (adult-child–spouse)
3: Are adult-children and spousal caregivers’ decisions influenced / helped by different individuals and information sources?	Binary logistic			

The dependent variables estimated in questions two and three are based on a specific decision. Therefore, to measure potential differences in adult-child and spousal caregivers’ experiences by the type of decision (e.g., planning treatment, challenging medical authority), we estimated a second set of logistic regression models with an interaction term between caregiver-patient relationship and the decision type covariate. Again, to aid interpretation, we used STATA’s margins command to present differences in adult-children’s and spouses’s probability of the outcome for each decision type.

All logistic regression models adjusted for caregiver’s gender, race, Hispanic ethnicity, and educational attainment. Since patients’ capacity to communicate with their medical team could influence a caregiver’s involvement in decisions, we also adjusted for how effectively a patient communicated with their oncology doctor. Because these covariates had low levels of missingness (<1.6%), we used listwise deletion. We did not adjust for other factors because they are highly correlated with being an adult-child or spousal caregiver (e.g., caregiver age, employment). All analyses were conducted using STATA17. Alpha level for significance was set at 0.05.

## Results

Compared to spouses, adult-children were younger, had greater educational attainment, were more frequently employed, had higher household incomes, and a greater share lived in rural areas (Table 2). Adult-children’s care recipients were older on average, more likely to have breast cancer and less likely to have prostate cancer, and adult-children reported that their parents communicated less effectively with a medical provider compared to spousal caregivers’ spouses with cancer. Adult-children also reported spending less time caregiving than spousal caregivers and reported the involvement of multiple caregivers more frequently.

**Table 2:** Summary statistics (N = 1206).

		Spouse (n = 314)	Adult-child (n = 892)	
Measures	Missing	Percent	Percent	P
*Caregiver*				
Gender	19			
Female		61.4	57.6	0.25
Male		38.6	42.4	
Age	3			
18–24		1.27	6.07	<0.001
25–34		10.83	22.61	
35–44		18.79	33.30	
45–54		17.20	17.55	
55–64		21.97	16.65	
65–74		23.57	3.71	
75 or older		6.37	0.11	
Education	1			
High school/GED or less		15.61	9.33	<0.001
Some college / Associate degree		27.39	20.67	
College		34.08	42.58	
Graduate school		22.93	27.42	
Employment	5			
Retired/unemployed & not looking for work		36.66	12.40	<0.001
Part-time/student/unemployed & looking for work		16.72	18.70	
Full-time		46.62	69.00	
Race	0			
White		82.32	80.38	0.60
Black		10.29	10.60	
Asian		3.86	6.76	
All else		3.54	2.25	
Ethnicity (ref = non-Hispanic)	0			
Hispanic		13.46	16.40	0.22
Rurality (ref = urban)	0			
Rural		21.09	15.76	0.03
*Patient*				
Gender	22			
Female		41.26	61.16	<0.001
Male		59.74	39.84	
Age	3			
18–24		1.28	0.45	<0.001
25–34		8.31	0.67	
35–44		12.78	1.69	
45–54		16.29	9.44	
55–64		20.45	27.30	
65–74		27.80	30.67	
75 or older		13.10	29.78	
Cancer stage (ref = stage I/II)	175			
Stage III / IV		57.45	55.24	0.55
Cancer type	5			
Breast		16.99	25.98	0.00
Prostate		21.15	13.72	
Lung		10.58	13.50	
Colon/rectal		6.09	7.54	
Other		45.19	39.26	
Hours providing support / day	10			
Less than 1 hour		13.55	3.60	<0.001
1–2 hours		25.80	33.75	
3–4 hours		21.94	34.65	
5–6 hours		14.84	14.33	
7–10 hours		13.23	8.01	
More than 10 hours		10.65	5.64	
Number of caregivers (ref = sole caregiver)	5			
Only caregiver		66.35	36.49	<0.001
1 other caregiver		20.51	43.53	
2 other caregivers		11.54	15.97	
3 or more other caregivers		1.60	4.05	
Caregiver and patient live together (ref = no)	0			
Yes		87.90	38.50	<0.001
How often patient can communicate with doctor effectively	8			
Never / Not usually / Sometimes		28.85	38.83	0.002
Most of the time / All of the time		71.15	61.17	

### Question 1: Are adult-children involved more frequently in certain types of decisions than spousal caregivers?

Chi-square tests demonstrated that adult-child and spousal caregivers had similar frequencies of involvement in each decision type with the following exceptions: spouses reported being involved in the treatment plan decision more often than adult-child caregivers (65.4% vs. 56.3%; p = 0.005) while adult-child caregivers reported participating more often in decisions to seek a second opinion (49.3% vs. 41.7%; p = 0.02), alternative treatments (37.6% vs. 27.2%; p = 0.001), and hospice (27.7% vs. 20.5%; p = 0.01) (see S1A Fig). When comparing involvement by decision type, adult-children were more frequently involved in decisions to challenge medical authority (72.8% vs. 61.2%; p<0.001). In models adjusting for covariates (gender, race, Hispanic ethnicity, educational attainment, patient ability to communicate with oncologist), adult-children were no longer statistically more likely to be involved in second opinions than spouses (beta = 0.26, p = 0.06), but were statistically less likely to be involved in at least one planning decision (beta = -0.41, p = 0.04) (Table 3).

**Table 3:** Adjusted log-odds of caregiver being involved in different decisions by relation to patient (N = 1,171)*.

Decision	Log-odds (ref = spouse)	95% CI	P
*Planning treatment*			
Whether to begin treatment	-0.24	-0.50, 0.03	0.09
Where to get treatment	-0.15	-0.43, 0.13	0.30
Treatment plan	-0.46	-0.74, -0.18	0.00
*Challenging medical authority*			
Whether to get second opinion on treatment	0.26	-0.01, 0.53	0.06
Whether to switch to another doctor/cancer center	0.08	-0.19, 0.36	0.55
To get alternative treatment	0.42	0.12, 0.71	0.01
*Assessing time-sensitive medical situation*			
Whether to go to emergency department	0.02	-0.25, 0.29	0.87
Which medications to take to manage symptoms	0.01	-0.26, 0.29	0.92
*Non-classified*			
To get biomarker test	0.15	-0.18, 0.38	0.38
Whether to be in a clinical trial	-0.01	-0.34, 0.32	0.97
To get palliative care	-0.02	-0.35, 0.31	0.92
To get rehabilitative services	0.09	-0.23, 0.42	0.56
Whether to stop cancer treatment	0.06	-0.25, 0.36	0.71
To have hospice	0.36	0.03, 0.68	0.03
*Grouped decision types*			
Planning treatment	-0.41	-0.81, -0.01	0.04
Challenging medical authority	0.50	0.22, 0.78	0.00
Assessing time-sensitive medical situation	0.04	-0.24, 0.32	0.78

^*^Covariates include caregiver’s gender, race, Hispanic ethnicity, and educational attainment, and patient ability to communicate with oncologist.

### Question 2: Does the primary decision-maker(s) differ when an adult-child is the caregiver versus a spouse?

Caregivers were asked which decision they remembered most clearly and who made the decision. Based on chi-square tests, across all decisions, adult-children and spouses sorted into different decision-maker roles at different rates (p = 0.01); relatively more adult-children than spouses reported acting as the primary decision-maker (17.2% vs. 11.2%; p = 0.01) while more spouses reported making a joint decision with the patient (55.8% vs. 45.1%; p = 0.001) (S1A Table). In adjusted models, adult-children were no longer more likely than spouses to act as primary decision-maker, but became significantly more likely than spouses to identify the patient as primary decision-maker (5.6 percentage points greater probability; p = 0.02), largely due to non-classified types of decisions (Table 4). Adult-children were more likely to act as primary decision-maker for decisions regarding planning treatment (7.2 percentage points greater probability; p = 0.01) and challenging medical authority (12.6 percentage points; p<0.001). Spouses’ greater probability of reporting that they made a joint decision with the patient was largely due to planning treatment decisions (12.4 percentage points greater; p = 0.01).

**Table 4:** Difference between adult-child versus spousal caregivers’ adjusted predicted probability of primary decision-maker by type of decision (N = 1,152).

	Patient made it		I made it		We made it together		Clinical team		Multiple actors/groups	
Decision type	Difference^1^ (95% CI)	P	Difference^1^ (95% CI)	P	Difference^1^ (95% CI)	P	Difference^1^ (95% CI)	P	Difference^1^ (95% CI)	P
All decisions^2^	5.60%	0.02	5.26%	0.05	-13.15%	0.00	0.52%	0.80	1.77%	0.44
	(0.01, 10.43)		(0.00, 10.56)		(-19.64, -6.67)		(-3.52, 4.57)		(-2.67, 6.21)	
Planning treatment (begin tx, where to receive tx, tx plan)^3^	1.90%	0.49	7.23%	0.01	-12.38%	0.01	0.50%	0.85	2.74%	0.31
	(-3.45, 7.26)		(1.60, 8.89)		(-20.97, -3.80)		(-4.71, 5.71)		(-2.52, 8.01)	
Challenging medical authority (2^nd^ opinion, switching md/center, alternative tx)^3^	7.49%	0.28	12.59%	0.00	-15.56%	0.10	1.16%	0.84	-5.68%	0.42
	(-5.98, 20.96)		(7.457, 17.72)		(-34.33, 3.21)		(-10.41, 12.74)		(-19.39, 8.04)	
Assessing medical situation (emergency dept, meds for sx)^3^	-0.01%	1.00	2.28%	0.75	-12.92%	0.13	7.10%	0.19	3.55	0.14
	(-7.46, 7.44)		(-11.66, 16.22)		(-29.73, 3.88)		(3.43, 17.62)		(-7.53, 14.63)	
All else (clinical trial, biomarker test, palliative care, rehab services, hospice)^3^	13.28%	0.02	-3.06%	0.68	-10.17%	0.25	-3.86%	0.47	3.82%	0.50
	(1.85, 24.70)		(-17.68, 11.55)		(-27.53, 7.19)		(-14.36, 6.65)		(-7.20, 14.84)	

^1^Difference calculated as adult-child’s minus spouses’ predicted probability and is reported as percentage point difference. Positive values indicate adult-child have greater predicted probability of the outcome than spouses.

^2^Multinomial logit model with no interaction term.

^3^Multinomial logit model includes a patient-caregiver relationship x decision type interaction term

### Question 3: Are adult-children and spousal caregivers’ decisions differentially influenced by other individuals and information sources?

To investigate whether additional individuals or information sources differentially shaped adult-child and spousal caregivers’ decision-making, we examined: (1) how frequently others were involved in decision-making and (2) which individuals and institutions caregivers turned to for help and information to make decisions. Adult-children reported more often than spouses that other family members and friends were involved in decisions (39.7% vs. 22.3%; p<0.001) (S2A Fig). Adjusted models (Table 5) indicated this difference was primarily driven by differences in decisions about treatment planning (21.7 percentage points; p<0.001) and time-sensitive medical situations (19.6 percentage points; p = 0.01). Models also indicated spiritual counselors were more likely to be involved in spouses’ decisions whether to challenge medical authority (14.3 percentage points; p = 0.046) while adult-children were more likely to have spiritual counselors involved in decisions assessing emergent situations (7.8 percentage points; p = 0.03).

**Table 5:** Difference between adult-child and spousal caregivers’ adjusted predicted probabilities of others’ decision-making involvement by type of decision (N = 1171)*.

	Patient		Family/friends		Spiritual/religious counselor		Oncology team		Non-oncology provider	
Decision type	Difference^1^ (95% CI)	P	Difference^1^ (95% CI)	P	Difference^1^ (95% CI)	P	Difference^1^ (95% CI)	P	Difference^1^ (95% CI)	P
All decisions^2^	2.31%	0.45	18.25%	0.00	-1.89%	0.26	-5.13%	0.12	0.85%	0.72
	(-3.73, 8.36)		(11.70, 24.80)		(-5.15, 1.38)		(-11.54, 1.28)		(-3.76, 5.46)	
Planning treatment (begin tx, where to receive tx, tx plan)^3^	1.21%	0.77	21.66%	0.00	-4.00%	0.10	-6.13%	0.10	1.24%	0.68
	(-6.80, 9.22)		(14.48, 28.86)		(-8.76, 0.75)		(-13.49, 1.22)		(-4.63, 7.11)	
Challenging medical authority (2^nd^ opinion, switching md/center, alternative tx)^3^	4.37%	0.62	-8.49%	0.38	-14.25%	0.046	-6.55%	0.50	1.91%	0.77
	(-12.73, 21.47)		(-27.39, 10.42)		(-28.23, -0.27)		(-25.38, 12.28)		(-10.95, 14.76)	
Assessing time-sensitive medical situation (emergency dept, meds for sx)^3^	-2.29%	0.78	19.60%	0.01	7.81%	0.03	12.61%	0.14	9.47%	0.09
	(-18.11, 13.52)		(4.72, 34.47)		(0.82, 14.80)		(-3.93, 29.16)		(-1.30, 20.25)	
All else (clinical trial, biomarker test, palliative care, rehab services, hospice)^3^	9.09%	0.30	14.38%	0.08	4.19%	0.37	-1.88%	0.83	-10.30%	0.14
	(-8.17, 26.35)		(-1.55, 30.31)		(-4.98, 13.37)		(-19.10, 15.34)		(-24.03, 3.43)	

*Covariates include caregiver’s gender, race, Hispanic ethnicity, and educational attainment, and patient ability to communicate with oncologist.

^1^Difference calculated as adult-child’s minus spouses’ predicted probabilities and is reported as percentage point difference. Positive values indicate adult-child have greater probability of the outcome than spouses.

^2^Binary logistic model with no interaction term.

^3^Binary logistic model includes a patient-caregiver relationship x decision type interaction term.

Regarding sources of information and help, chi-square tests suggested that similar proportions of adult-children and spouses used certain types of resources (e.g., internet, education materials, social media), but that adult-children turned to the oncology team (57.2% vs. 48.7%; p = 0.001) and friends and family (43.8% vs. 34.7%; p = 0.005) more often than spouses did and that more spouses than adult-children reported not seeking *any* help or information (7.0% vs. 3.9%; p = 0.03) (Table 6; for full results, see S2A Table). Adjusted models indicated that these differences were largely due to decisions assessing medical situations (oncology team: 18.5 percentage points, p = 0.03; friends or family: 18.9 percentage points, p = 0.02; did not seek info: -12.4 percentage points, p = 0.02). Models demonstrated several other statistically significant differences by decision type for other types of resources (see S2B Table).

**Table 6:** Adult-child and spousal caregivers’ frequency and difference in adjusted predicted probability of others’ decision-making involvement by type of decision.

Relation to patient (N = 1206)^1^	Oncology team		Family/friends		Did not seek info	
	%	P	%	P	%	P
Adult-child	57.17	0.001	43.83	0.005	3.92	0.03
Spouse	48.73		34.71		7.01	
**Decision type (N = 1171)** ^**2**^	**Difference**^**3**^ **(95% CI)**	**P**	**Difference**^**3**^ **(95% CI)**	**P**	**Difference**^**3**^ **(95% CI)**	**P**
All decisions^4^	8.42%	0.01	7.91%	0.02	-2.26%	0.08
	(1.98, 14.87)		(1.34, 14.48)		(-4.82, 0.30)	
Planning treatment (begin tx, where to receive tx, tx plan)^5^	6.87%	0.12	6.82%	0.11	1.25%	0.38
	(-1.69, 15.43)		(-1.61, 15.24)		(-1.51, 4.00)	
Challenging medical authority (2^nd^ opinion, switching md/center, alternative tx)^3^	16.49%	0.05	-8.00%	0.41	0.38%	0.93
	(-0.10, 33.07)		(-26.91, 10.90)		(-8.07, 8.82)	
Assessing medical situation (emergency dept, meds for sx)^5^	18.54%	0.03	18.91%	0.02	-12.43%	0.02
	(1.94, 35.13)		(3.39, 34.44)		(-23.23, -1.62)	
All else (clinical trial, biomarker test, palliative care, rehab services, hospice)^5^	10.92%	0.22	9.34%	0.28	-6.51%	0.15
	(-6.50, 28.35)		(-7.74, 26.42)		(-15.32, 2.29)	

^1^Chi-square test results

^2^Logistic model adjusted for caregiver’s gender, race, Hispanic ethnicity, and educational attainment and patient’s ability to communicate with oncologist

^3^Difference calculated as adult-child’s minus spouses’ predicted probabilities and is reported as percentage point difference. Positive values indicate adult-child have greater predicted probabilities of the outcome than spouses.

^4^Binary logistic model with no interaction term

^5^Binary logistic model includes a patient-caregiver relationship x decision type interaction term

## Discussion

This study explores how a caregiver’s relation to the patient and thus their social position (e.g., child, spouse, friend) shapes medical decision-making. Our analysis of a sample of over 1,200 caregivers compares adult-child and spousal cancer caregivers’ decision-making experiences, including the types of decisions they participate in, who acts as the primary decision-maker, and the resources they utilize to inform their decisions. Our results indicate that many aspects of decision-making are similar for adult-child and spousal caregivers and yet that there are some notable differences: First, spouses were more likely to be involved in treatment planning decisions, such as what type of treatment to receive. In contrast, more adult-children reported participating in decisions about whether to challenge medical authority, such as seeking alternative treatment. These differences suggest that many adult-children are not involved until considerably after diagnosis since treatment planning decisions are typically the first types that patients and caregivers are confronted with while decisions to seek alternative options often come later in the treatment trajectory. Second, our results indicate that adult-children and spouses, and subsequently patients, have different roles in medical decision-making, particularly treatment planning decisions. Like Ozdemir et al. (2022), [21] we also found that adult-children were more likely than spouses to report that the patient was the primary decision-maker and that they acted as the primary decision-maker regarding treatment and challenging medical authority decisions. Conversely, spouses were more likely than adult-children to report jointly making decisions with patients. Third, adult-children were more likely than spouses to rely on others, particularly family members and friends and the oncology team, for additional decision support including help making the decision and as an information source.

Consistent with prior research, we found that caregivers are highly involved in patient medical decision-making and that their involvement varies [2, 8, 22, 23]. We build on prior work by finding evidence that the caregiver-patient relation partially explains why involvement varies and for what decisions. Our work helps situate and support the findings of a small set of studies, typically of treatment decisions, that compare the involvement of adult-children to other caregivers-patient relations. These studies find that, compared to other caregivers, adult-children possess different perspectives on decision-making (e.g., prefer greater involvement than patients do) and are more likely to seek information [11, 12, 24]. In conjunction with these studies, our findings indicate that a caregiver’s relation to the patient shapes many decision factors that could have consequences for patient care and friends’ and families’ care experiences. Future research should explore these potential consequences. For instance, research should investigate whether adult-children’s and spouses’ differences in probabilities of primary decision-makers influences the extent that patients’ goals of care are met or caregivers’ bereavement.

To date, most cancer caregiver interventions have been designed for patients’ spouses/partners [25]. However, our results suggest that adult-children and spouses require different levels and types of support [26]. Thus interventions may better support caregivers (and therefore patients) if we appropriately tailor interventions based on caregivers’ relationship to patient. This may be particularly relevant to sharing information, which is fundamental to all caregivers’ decision-making [1, 27], especially cancer caregivers [28]. Our finding that patients frequently do not involve their adult-children until later in the diagnosis suggests that adult-child and spousal caregivers need different amounts of information at different time points. For instance, once involved, adult-child caregivers may need extra support to quickly adjust to their new roles. Similarly, adult-children were more likely to act as primary decision-maker for certain decision types, a weighty responsibility that requires preparation and support. In our study, spouses were also less likely than adult-children to seek information or help or from certain valuable sources, like the oncology team and friends and family, particularly decisions assessing medical situations. One potential explanation is that spouses may be more hesitant than adult-children to seek information and support. In parallel, the evidence may also suggest that adult-child caregivers are less familiar than spouses with the patient’s preferences for care and rely on others to help supplement their knowledge and to help spread the decision-making ownership and responsibilities. Both explanations highlight the potential benefit of communication coaching and tools, such as question prompt lists, to optimize information gathering and coalition building [29, 30]. Adult-children’s increased informational needs may also drive their greater probability to be involved in decisions that challenge medical authority, since seeking new or alternative options can be a reaction to a poor prognosis [31] or a result of receiving conflicting information from clinicians [32, 33]. If so, potentially ill-advised decisions may be prevented by including adult-child caregivers in prognostis discussions with clear and consistent messaging from clinicians. To design an effective solution, research, particularly qualitative, is needed to explore these differences in information and help seeking to identify which population group is in need and why.

Findings are limited by several potential issues related to survey design. First, we could not estimate whether differences in how frequently caregivers were involved with decisions was because a decision was not applicable to the patient or had yet to be encountered. This could have particularly affected results regarding decisions typically faced later such as clinical trial participation and hospice. Second, the survey did not measure actual decisions made and/or their potential effect on patient outcomes. For instance, our findings suggest that adult-child caregivers are more likely than spouses to participate in decisions to potentially challenge medical authority (e.g., switch oncologists or pursue alternative treatments), but we cannot assess whether adult-children are ultimately more likely to decide to challenge that authority. Future studies should explore caregiver’s relationship to the patient, their involvement in patient’s medical decision making, and its effect on outcomes. Third, while the sample is diverse, it is not nationally representative, limiting ability to generalize findings. Similarly, since this sample was derived from market panels and administered online, selection bias likely occurred across urban-rural, income, age, and race and ethnic lines [34]. Sampled caregivers reported higher levels of educational attainment compared to nationally representative surveys of cancer caregivers [7], may be partly due to the survey’s restriction to respondents who specifically reported participating in health-related decision-making for the patient.

Due to collinearity, we also could not explore the extent that cohort versus relationship effects drove experiences. Since younger generations have more experience with the concept of shared decision-making with providers [35], this could have been most relevant to findings that adult-children were more likely than spouses to be involved in decisions to challenge medical authority. Similarly, the reader might notice we did not adjust for many factors that can affect decision-making (e.g., sole or distance caregiver). This decision was intentional since many of these factors are intrinsic to the social position of being a patient’s adult-child or spouse. For instance, spouses are more likely to co-reside than parents and their adult caregivers. This fact likely contributes to our finding that spouses are more likely than adult-children to participate in treatment planning and so adjusting for this variable would be inappropriate [36]. Similarly, our finding that friends and family members are more often involved in decision-making when the caregiver is an adult-child is likely partly explained by adult-children’s increased probability to share caregiving responsibilities with others. Yet, there are many sociocultural reasons for this fact that are intrinsic to the child position; patients typically have multiple children, and they are likely to be consulted for significant healthcare decisions [37–39]. Additionally, for many families, caregiving is a communal affair to some extent where others are involved and consulted [40]. Thus, adjusting for these factors raises substantive questions about what types of adult-child and spousal populations we would be comparing while weakening the explanatory power of our models. Future analyses could limit their analyses to adult-children to disentangle relationship from care and familial context. This paper’s goal, however, is to describe ways to support different types of caregivers who represent different caregiving contexts rather than understand unmalleable factors.

## Conclusion

Family caregivers are critical to medical decision-making in cancer care, which is often deeply uncertain, complex, and highly stressful for caregivers and patients. Caregivers possess varied aptitudes for decision-making based on their life experience, resources, and relation to the patient and thus may require different types and levels of decision-making support. Yet, little research has explored how caregiving experiences differ by the caregiver-patient relationship. Our study indicates that how caregivers are related to patients–specifically whether they are the patient’s child vs spouse–has important consequences for which medical decisions caregivers influence, their level of influence, and what resources they draw on to inform the decisions. These findings highlight the need for further research to identify the ways that different patient-caregiver relations may influence medical decisions and patient care. Our results also suggest that efforts to support caregivers’ and patients’ decision-making could, at the very least, be tailored to the distinct social relations that connect caregivers and patients and associated decision support needs.

## Supporting information

S1 Fig Percent of caregivers involved in decisions by relation to patient (N = 1206). (DOCX)

S2 Fig Frequency of others’ involvement in decision-making by caregiver’s relation to patient (N = 1206). (DOCX)

S1 Table Frequency of primary decision-maker by relation to patient (N = 1185). (DOCX)

S2 Table **A**. Frequency of caregivers who use different sources of help and info by relation to patient (N = 1206). **B**. Difference between adult-child and spousal caregivers’ adjusted predicted probabilities of others’ decision-making involvement by type of decision (N = 1171).
(ZIP)

S1 File (DOCX)
