# Supplementary material for: A comparison of adult-child and spousal cancer caregivers’ participation in medical decisions
Source: PLoS One. 2024 Jun 13;19(6):e0300450. doi: 10.1371/journal.pone.0300450 (PMC11175391; doi:10.1371/journal.pone.0300450)
Supplement: S2 Fig — (DOCX) [file pone.0300450.s002.docx]

**Figure 2A.** Frequency of others’ involvement in decision-making by caregiver’s relation to patient (N=1206)

*= p<0.05; **=p<0.01; ***=p<0.001
