# Supplementary material for: A comparison of adult-child and spousal cancer caregivers’ participation in medical decisions
Source: PLoS One. 2024 Jun 13;19(6):e0300450. doi: 10.1371/journal.pone.0300450 (PMC11175391; doi:10.1371/journal.pone.0300450)
Supplement: S1 Table — (DOCX) [file pone.0300450.s003.docx]

| **Table 1A.** Frequency of primary decision-maker by relation to patient (N=1185) | | | |
| --- | --- | --- | --- |
| **Decision maker** | **Spouse / partner**  **(N=312)** | **Adult-child**  **(N=873)** | **P** |
| Patient made it | 11.54% | 14.78% | 0.16 |
| I made it | 11.22% | 17.18% | 0.01 |
| We made it together | 55.77% | 45.13% | 0.001 |
| Clinical team | 10.58% | 10.54% | 0.99 |
| Multiple actors/groups | 10.90% | 12.37% | 0.49 |
| *Global test* |  |  | 0.01 |
