# Supplementary material for: A comparison of adult-child and spousal cancer caregivers’ participation in medical decisions
Source: PLoS One. 2024 Jun 13;19(6):e0300450. doi: 10.1371/journal.pone.0300450 (PMC11175391; doi:10.1371/journal.pone.0300450)
Supplement: S2 Table — A. Frequency of caregivers who use different sources of help and info by relation to patient (N = 1206). B. Difference between adult-child and spousal caregivers’ adjusted predicted probabilities of others’ decision-making involvement by type of decision (N = 1171). (ZIP) [file pone.0300450.s004.zip › S2B_Table.pdf]

Table 2B. Difference between adult-child and spousal caregivers' adjusted predicted probabilities of others' decision-making involvement by type of decision (N=1171)*

Decision type	Oncology team		Family/friends		Non-oncology provider		Internet		Social media	
	Difference[1] (95% CI)	P	Difference[1] (95% CI)	P	Difference[1] (95% CI)	P	Difference[1] (95% CI)	P	Difference[1] (95% CI)	P
All decisions[2]	8.42% (1.98, 14.87)	0.01	7.91% (1.34, 14.48)	0.02	6.16% (-0.44, 12.75)	0.07	-0.04% (-6.38, 6.30)	0.99	-3.21% (-7.41, 0.99)	0.14
Planning treatment (begin tx, where to receive tx, tx plan)[3]	6.87% (-1.69, 15.43)	0.12	6.82% (-1.61, 15.24)	0.11	4.24% (-4.41, 12.89)	0.34	-0.47% (-8.96, 8.02)	0.91	-6.48% (-12.64, -0.32)	0.04
Challenging medical authority (2nd opinion, switching md/center, alternative tx)[3]	16.49% (-0.10, 33.07)	0.05	-8.00% (-26.91, 10.90)	0.41	5.46% (-13.36, 24.28)	0.57	-2.87 (-21.61, 15.87)	0.76	-4.02 (-17.97, 9.93)	0.57
Assessing medical situation (emergency dept, meds for sx)[3]	18.54% (1.94, 35.13)	0.03	18.91% (3.39, 34.44)	0.02	18.58% (3.16, 34.00)	0.02	12.47% (-1.86, 26.81)	0.09	-4.69% (-16.81, 7.43)	0.45
All else (clinical trial, biomarker test, palliative care, rehab services, hospice)[3]	10.92% (-6.50, 28.35)	0.22	9.34% (-7.74, 26.42)	0.28	0.76% (-16.24, 17.76)	0.93	-9.09% (-25.34, 7.15)	0.27	10.41 (1.23, 19.59)	0.03

Decision type	Patient education		Government agencies		Non-profits		Did not seek info	
	Difference[1] (95% CI)	P	Difference[1] (95% CI)	P	Difference[1] (95% CI)	P	Difference[1] (95% CI)	P
All decisions[2]	-2.58% (-8.83, 3.67)	0.42	0.80% (-3.03, 4.68)	0.67	0.64% (-4.10, 5.38)	0.79	-2.26% (-4.82, 0.30)	0.08
Planning treatment (begin tx, where to receive tx, tx plan)[3]	-6.25% (14.63, 2.14)	0.14	-0.75% (-5.74, 4.24)	0.77	-1.06% (-7.23, 5.12)	0.74	1.25% (-1.51, 4.00)	0.38
Challenging medical authority (2nd opinion, switching md/center, alternative tx)[3]	10.76% (28.77, 7.24)	0.24	-2.56 (-13.40, 8.28)	0.64	4.82% (-8.53, 18.17)	0.48	0.38% (-8.07, 8.82)	0.93
Assessing medical situation (emergency dept, meds for sx)[3]	17.50% (2.25, 32.75)	0.02	2.28% (-5.75, 10.31)	0.58	3.15% (9.36, 15.66)	0.62	-12.43% (-23.23, -1.62)	0.02
All else (clinical trial, biomarker test, palliative care, rehab services, hospice)[3]	-1.32 (-18.60, 15.94)	0.88	7.76% (-1.25, 16.78)	0.09	2.35 (-8.01, 12.71)	0.66	-6.51% (-15.32, 2.29)	0.15

[*]Covariates include caregiver's gender, race, Hispanic ethnicity, and educational attainment, and patient ability to communicate with oncologist.

[1]Difference calculated as adult-child's minus spouses' predicted probabilities and is reported as percentage point difference. Positive values indicate adult-child have greater probability of the outcome than spouses.

[2]Binary logistic model with no interaction term.

[3]Binary logistic model includes a patient-caregiver relationship x decision type interaction term.
