# Supplementary material for: A comparison of adult-child and spousal cancer caregivers’ participation in medical decisions
Source: PLoS One. 2024 Jun 13;19(6):e0300450. doi: 10.1371/journal.pone.0300450 (PMC11175391; doi:10.1371/journal.pone.0300450)
Supplement: S2 Table — A. Frequency of caregivers who use different sources of help and info by relation to patient (N = 1206). B. Difference between adult-child and spousal caregivers’ adjusted predicted probabilities of others’ decision-making involvement by type of decision (N = 1171). (ZIP) [file pone.0300450.s004.zip › S2A_Table.pdf]

Table 2A. Frequency of caregivers who use different sources of help and info by relation to patient (N=1206)

**Info source**	**Percent** Spouse / partner	Adult-child	**P**
Oncology team	48.73%	57.17%	0.01
Friends or family	34.71%	43.83%	0.005
Non-oncology provider	38.54%	44.73%	0.06
Internet	34.08%	35.54%	0.64
Patient education given to us by the patient's care team	34.08%	33.63%	0.89
Social Media	14.97%	12.33%	0.23
Government agencies or organizations	7.64%	9.08%	0.44
Non-profit organization for caregiving or cancer	13.69%	14.57%	0.70
Never looked for help or information	7.01%	3.92%	0.03
