# Supplementary material for: A comparison of adult-child and spousal cancer caregivers’ participation in medical decisions
Source: PLoS One. 2024 Jun 13;19(6):e0300450. doi: 10.1371/journal.pone.0300450 (PMC11175391; doi:10.1371/journal.pone.0300450)
Supplement: S1 File — (DOCX) [file pone.0300450.s005.docx]

# Supporting Information

**Figure 1A.** Percent of caregivers involved in decisions by relation to patient (N=1206)

*= p<0.05; **=p<0.01; ***=p<0.001

| Table 1A. Frequency of primary decision-maker by relation to patient (N=1185) | | | |
| --- | --- | --- | --- |
| **Decision maker** | **Spouse / partner**  **(N=312)** | **Adult-child**  **(N=873)** | **P** |
| Patient made it | 11.54% | 14.78% | 0.16 |
| I made it | 11.22% | 17.18% | 0.01 |
| We made it together | 55.77% | 45.13% | 0.001 |
| Clinical team | 10.58% | 10.54% | 0.99 |
| Multiple actors/groups | 10.90% | 12.37% | 0.49 |
| *Global test* |  |  | 0.01 |

**Figure 2. Frequency of others’ involvement in decision-making by caregiver’s relation to patient (N=1206)**

*= p<0.05; **=p<0.01; ***=p<0.001

| **Table 2A.** Frequency of caregivers who use different sources of help and info by relation to patient (N=1206) | | | |
| --- | --- | --- | --- |
|  | **Percent** | | |
| **Info source** | **Spouse / partner** | **Adult-child** | **P** |
| Oncology team | 48.73% | 57.17% | 0.01 |
| Friends or family | 34.71% | 43.83% | 0.005 |
| Non-oncology provider | 38.54% | 44.73% | 0.06 |
| Internet | 34.08% | 35.54% | 0.64 |
| Patient education given to us by the patient's care team | 34.08% | 33.63% | 0.89 |
| Social Media | 14.97% | 12.33% | 0.23 |
| Government agencies or organizations | 7.64% | 9.08% | 0.44 |
| Non-profit organization for caregiving or cancer | 13.69% | 14.57% | 0.70 |
| Never looked for help or information | 7.01% | 3.92% | 0.03 |

| **Table 2B.** Difference between adult-child and spousal caregivers' adjusted predicted probabilities of others' decision-making involvement by type of decision (N=1171)* | | | | | | | | | | | |  |  |  |  |  |
| --- | --- | --- | --- | --- | --- | --- | --- | --- | --- | --- | --- | --- | --- | --- | --- | --- |
|  | **Oncology team** | | **Family/friends** | | **Non-oncology provider** | | **Internet** | | **Social media** | | |  |  |  |  |  |
| **Decision type** | **Difference^1^ (95% CI)** | **P** | **Difference^1^ (95% CI)** | **P** | **Difference^1^ (95% CI)** | **P** | **Difference^1^ (95% CI)** | **P** | **Difference^1^ (95% CI)** | **P** | |  |  |  |  |  |
| All decisions^2^ | 8.42% | 0.01 | 7.91% | 0.02 | 6.16% | 0.07 | -0.04% | 0.99 | -3.21% | 0.14 | |  |  |  |  |  |
|  | (1.98, 14.87) |  | (1.34, 14.48) |  | (-0.44, 12.75) |  | (-6.38, 6.30) |  | (-7.41, 0.99) |  | |  |  |  |  |  |
| Planning treatment (begin tx, where to receive tx, tx plan)^3^ | 6.87% | 0.12 | 6.82% | 0.11 | 4.24% | 0.34 | -0.47% | 0.91 | -6.48% | 0.04 | |  |  |  |  |  |
|  | (-1.69, 15.43) |  | (-1.61, 15.24) |  | (-4.41, 12.89) |  | (-8.96, 8.02) |  | (-12.64, -0.32) |  | |  |  |  |  |  |
| Challenging medical authority (2^nd^ opinion, switching md/center, alternative tx)^3^ | 16.49% | 0.05 | -8.00% | 0.41 | 5.46% | 0.57 | -2.87 | 0.76 | -4.02 | 0.57 | |  |  |  |  |  |
|  | (-0.10, 33.07) |  | (-26.91, 10.90) |  | (-13.36, 24.28) |  | (-21.61, 15.87) |  | (-17.97, 9.93) |  | |  |  |  |  |  |
| Assessing medical situation (emergency dept, meds for sx)^3^ | 18.54% | 0.03 | 18.91% | 0.02 | 18.58% | 0.02 | 12.47% | 0.09 | -4.69% | 0.45 | |  |  |  |  |  |
|  | (1.94, 35.13) |  | (3.39, 34.44) |  | (3.16, 34.00) |  | (-1.86, 26.81) |  | (-16.81, 7.43) |  | |  |  |  |  |  |
| All else (clinical trial, biomarker test, palliative care, rehab services, hospice)^3^ | 10.92% | 0.22 | 9.34% | 0.28 | 0.76% | 0.93 | -9.09% | 0.27 | 10.41 | 0.03 | |  |  |  |  |  |
|  | (-6.50, 28.35) |  | (-7.74, 26.42) |  | (-16.24, 17.76) |  | (-25.34, 7.15) |  | (1.23, 19.59) |  | |  |  |  |  |  |
|  | **Patient education** | | **Government agencies** | | **Non-profits** | | **Did not seek info** | |  |  |  |  |  |  |  |  |
| **Decision type** | **Difference^1^ (95% CI)** | **P** | **Difference^1^ (95% CI)** | **P** | **Difference^1^ (95% CI)** | **P** | **Difference^1^ (95% CI)** | **P** |  |  |  |  |  |  |  |  |
| All decisions^2^ | -2.58% | 0.42 | 0.80% | 0.67 | 0.64% | 0.79 | -2.26% | 0.08 |  |  |  |  |  |  |  |  |
|  | (-8.83, 3.67) |  | (-3.03, 4.68) |  | (-4.10, 5.38) |  | (-4.82, 0.30) |  |  |  |  |  |  |  |  |  |
| Planning treatment (begin tx, where to receive tx, tx plan)^3^ | -6.25% | 0.14 | -0.75% | 0.77 | -1.06% | 0.74 | 1.25% | 0.38 |  |  |  |  |  |  |  |  |
|  | (14.63, 2.14) |  | (-5.74, 4.24) |  | (-7.23, 5.12) |  | (-1.51, 4.00) |  |  |  |  |  |  |  |  |  |
| Challenging medical authority (2^nd^ opinion, switching md/center, alternative tx)^3^ | 10.76% | 0.24 | -2.56 | 0.64 | 4.82% | 0.48 | 0.38% | 0.93 |  |  |  |  |  |  |  |  |
|  | (28.77, 7.24) |  | (-13.40, 8.28) |  | (-8.53, 18.17) |  | (-8.07, 8.82) |  |  |  |  |  |  |  |  |  |
| Assessing medical situation (emergency dept, meds for sx)^3^ | 17.50% | 0.02 | 2.28% | 0.58 | 3.15% | 0.62 | -12.43% | 0.02 |  |  |  |  |  |  |  |  |
|  | (2.25, 32.75) |  | (-5.75, 10.31) |  | (9.36, 15.66) |  | (-23.23, -1.62) |  |  |  |  |  |  |  |  |  |
| All else (clinical trial, biomarker test, palliative care, rehab services, hospice)^3^ | -1.32 | 0.88 | 7.76% | 0.09 | 2.35 | 0.66 | -6.51% | 0.15 |  |  |  |  |  |  |  |  |
|  | (-18.60, 15.94) |  | (-1.25, 16.78) |  | (-8.01, 12.71) |  | (-15.32, 2.29) |  |  |  |  |  |  |  |  |  |
| ^*^Covariates include caregiver’s gender, race, Hispanic ethnicity, and educational attainment, and patient ability to communicate with oncologist. | | | | | | | | | | |  |  |  |  |  |  |
| ^1^Difference calculated as adult-child's minus spouses' predicted probabilities and is reported as percentage point difference. Positive values indicate adult-child have greater probability of the outcome than spouses. | | | | | | | | | | |  |  |  |  |  |  |
| ^2^Binary logistic model with no interaction term. | | | | | | | | | | |  | |  |  |  |  |
| ^3^Binary logistic model includes a patient-caregiver relationship x decision type interaction term. | | | | | | | | | | |  |  |  |  |  |  |
